# Supplementary material for: Basin entropy: a new tool to analyze uncertainty in dynamical systems
Source: Sci Rep. 2016 Aug 12;6:31416. doi: 10.1038/srep31416 (PMC4981859; doi:10.1038/srep31416)
Supplement: Supplementary Information [file srep31416-s1.pdf]

Supplementary information for "Basin entropy: a new tool to analyze uncertainty in dynamical systems"

Alvar Daza,[1] Alexandre Wagemakers,[1] Bertrand Georgeot,[2]
David Guéry-Odelin,[3] and Miguel A.F. Sanjuán[1]

[1]*Nonlinear Dynamics, Chaos and Complex Systems Group, Departamento de Física, Universidad Rey Juan Carlos Tulipán s/n, 28933 Móstoles, Madrid, Spain*

[2]*Laboratoire de Physique Théorique, IRSAMC, Université de Toulouse, CNRS, UPS, France*

[3]*Laboratoire Collisions, Agrégats, Réactivité, IRSAMC, Université de Toulouse, CNRS, UPS, France*

PROOF OF THE LOG 2 CRITERION

The $\log 2$ criterion is a sufficient condition to prove the fractality of the basin boundaries. It is based on the concept of *boundary basin entropy*, defined as

$$S_{bb} = \frac{S}{N_b}, \tag{S.1}$$

where N_b is the number of boxes containing more than one color, that is, the number of boxes in the boundaries, and

$$S = \sum_{i=1}^{N} S_i = \sum_{i=1}^{N} \sum_{j=1}^{m_i} p_{i,j} \log \left(\frac{1}{p_{i,j}} \right). \tag{S.2}$$

Now we assume that the boundaries separating the basins are smooth. In this case, the number of boxes lying in the boundary separating two basins grows as

$$N_2 = n_2 \varepsilon^{-(D-1)}, \tag{S.3}$$

where D is the dimension of the phase space. For $D = 2$, the boundary would be a line, for $D = 3$, it would be a surface and so forth. However, there might be some boxes N_k lying in the boundaries of $k > 2$ different basins. These boxes are in the intersection of at least two subspaces of dimension $D - 1$, that is, they are in the intersection of two smooth boundaries. For instance, when $D = 2$, it simply means that two or more smooth curves intersect in a point or collection of points, and when $D = 3$, two or more smooth surfaces intersect forming smooth curves. Thus, the dimension of the subspace separating more than two basins must be $D - 2$, and the boxes N_k belonging to this subspace must grow as

$$N_k = n_k \varepsilon^{-(D-2)}. \tag{S.4}$$

Taking into account that the total number of boxes grows as $N = \tilde{n}\varepsilon^{-D}$, we can express N_2 in terms of N as

$$N_2 = n_2 \left(\frac{N}{\tilde{n}} \right)^{\frac{D-1}{D}}, \tag{S.5}$$

and for the boundary boxes separating more than two basins N_k, we have

$$N_k = n_k \left(\frac{N}{\tilde{n}} \right)^{\frac{D-2}{D}}. \tag{S.6}$$

At this point, we recall that the maximum possible value of S in a box with m different colors is $S = \log m$, which is the Boltzmann expression for the entropy of m equiprobable

microstates. Then, we can find that all the boxes in the boundary of two basins have $S \leqslant \log 2$, while for boxes in the boundary of k basins, $k > 2$, we have that $S \leqslant \log k$. Notice that the equality of the previous equations would be possible only in a pathological case where all the boxes in the boundaries have equal proportions of the different colors.

Then, the basin entropy S_{bb} for this hypothetical system with smooth boundaries is

$$S_{bb} \leq \frac{N_2 \log 2 + N_k \log k}{N_2 + N_k}. \tag{S.7}$$

By substituting N_2 and N_k by Eqs. S.5-S.6, we obtain the following expression

$$S_{bb} \leq \frac{n_2 \left(\frac{N}{\tilde{n}}\right)^{\frac{D-1}{D}} \log 2 + n_k \left(\frac{N}{\tilde{n}}\right)^{\frac{D-2}{D}} \log k}{n_2 \left(\frac{N}{\tilde{n}}\right)^{\frac{D-1}{D}} + n_k \left(\frac{N}{\tilde{n}}\right)^{\frac{D-2}{D}}}, \tag{S.8}$$

which can be simplified as

$$S_{bb} \leq \frac{n_2 N \log 2 + n_k \tilde{n} \log k}{n_2 N + n_k \tilde{n}}, \tag{S.9}$$

where $\tilde{n}, n_2, n_k$ are constants. Finally, we can take the limit of the previous inequality for a large number of boxes, that is when $N \to \infty$, leading to

$$\lim_{N \to \infty} S_{bb} \leq \log 2. \tag{S.10}$$

Therefore, we have proven that if the boundaries are smooth, then $S_{bb} \leq \log 2$, which is the same as to say that if $S_{bb} > \log 2$, then the boundaries are not smooth, i.e., they are fractal. This is what we call the $\log 2$ criterion.

This criterion is especially useful for experimental situations where the resolution cannot be arbitrarily chosen. In these cases we have a fixed value $\varepsilon > 0$. Nevertheless, if we take a sufficient large number of boxes N, then the $\log 2$ criterion holds. Moreover, the equality of Eq. S.10 never takes place, so that there is some room for the possible deviations caused by the impossibility of making an infinite number of simulations or experiments.

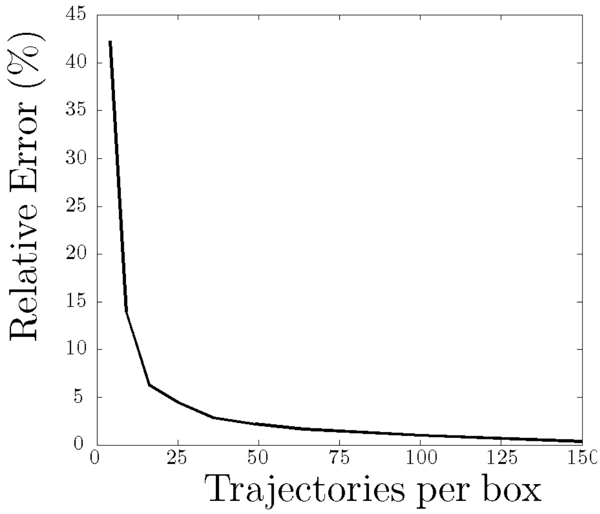

FIG. S.1. **Convergence of the basin entropy with the number of trajectories per box.** This figure represents the relative error in computing the basin entropy of Fig. 3-(d), taking as a reference the computation made with 2500 trajectories per box. In spite of the particularities of a given dynamical system (for instance the number of attractors is an important factor), we have seen that choosing 25 trajectories per box keeps the relative error below 5% in most cases and allows a fast computation. Therefore, the number of trajectories per box is a parameter that can be tuned in order to get accurate results in a short time.

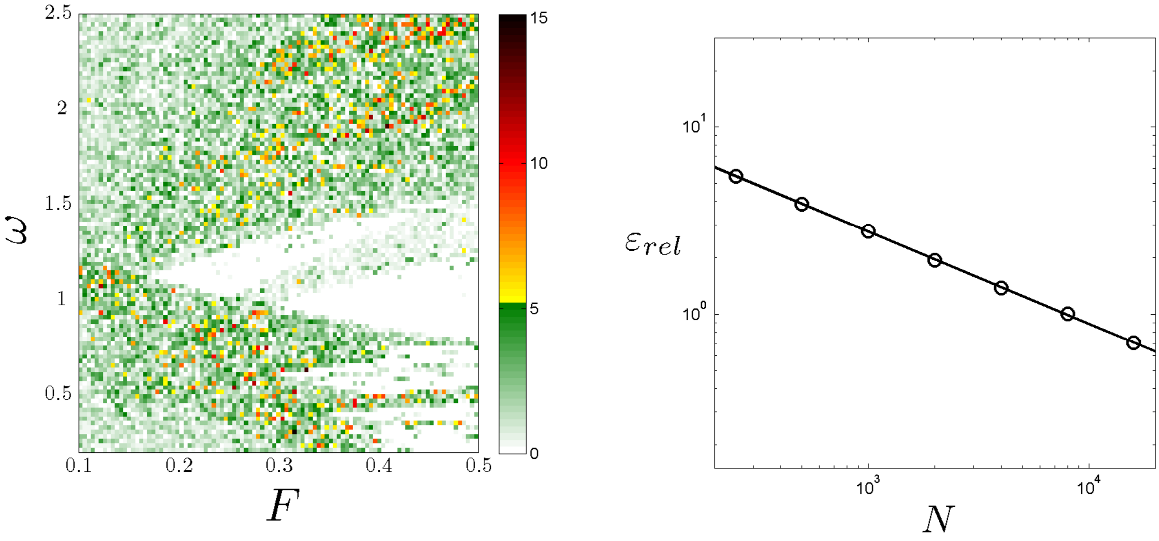

(a) (b)

FIG. S.2. **Random sampling error.** (a) This plot presents the relative error of the basin entropy estimation, that is $\frac{|S_b - S_b(RS)|}{\langle Sb \rangle} \times 100$, using 2000 boxes for the random sampling. The 94% of the times, the relative error is below 5%. (b) If a more precise value is needed this error decreases as $\frac{1}{\sqrt{N}}$.

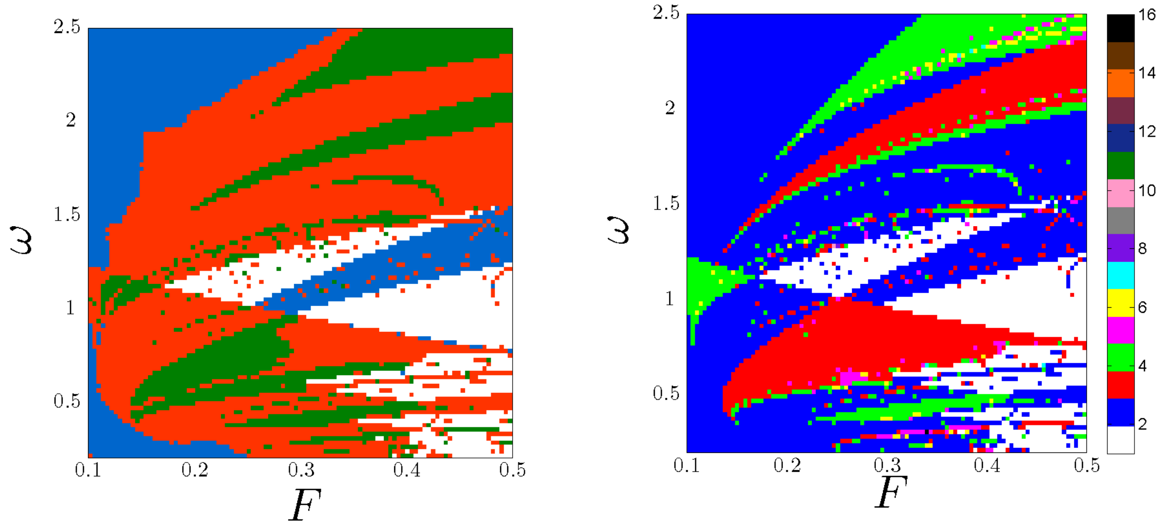

(a) (b)

FIG. S.3. **Some limitations to the log 2 criterion.** (a) White pixels indicate basins with one attractor, blue is for smooth boundaries, orange for fractal boundaries and green for fractal boundaries with $S_{bb} > \log 2$. All the basins with $S_{bb} > \log 2$ are fractal, but not all the fractal basins have $S_{bb} > \log 2$. The $\log 2$ criterion is a *sufficient but not necessary* condition for fractal boundaries. (b) Number of attractors in the parameter plane. The $\log 2$ criterion can only be fulfilled for basins with three or more attractors.
